# AKT1^E17K^‐Interacting lncRNA SVIL‐AS1 Promotes AKT1 Oncogenic Functions by Preferentially Blocking AKT1^E17K^ Dephosphorylation

**DOI:** 10.1002/advs.202500919

**Published:** 2025-03-26

**Authors:** Jingyi Wang, Wenying Chen, Qianying Li, Ruiyi Yang, Xiaorong Lin, Ping Han, Xiaoming Huang, Hai Hu, Man‐Li Luo

**Affiliations:** ^1^ Department of Otolaryngology‐Head and Neck Surgery Sun Yat‐Sen Memorial Hospital Sun Yat‐Sen University Guangzhou 510120 China; ^2^ Department of Gastroenterology Sun Yat‐Sen Memorial Hospital Sun Yat‐Sen University Guangzhou 510120 China; ^3^ Department of Oncology Sun Yat‐Sen Memorial Hospital Sun Yat‐sen University Guangzhou 510120 China; ^4^ Diagnosis and Treatment Center of Breast Diseases Shantou Central Hospital Shantou 515031 China; ^5^ Breast Cancer Center Zhejiang Cancer Hospital Hangzhou Institute of Medicine Chinese Academy of Sciences Hangzhou 310022 China; ^6^ Medical Research Center Sun Yat‐Sen Memorial Hospital Sun Yat‐Sen University Guangzhou 510120 China; ^7^ Guangdong Provincial Key Laboratory of Malignant Tumor Epigenetics and Gene Regulation Guangdong‐Hong Kong Joint Laboratory for RNA Medicine Sun Yat‐Sen Memorial Hospital Sun Yat‐Sen University Guangzhou 510120 China

**Keywords:** AKT1**^E17K^** mutation, lncRNA, PP2A, SVIL‐AS1

## Abstract

AKT1^E17K^ is a gain‐of‐function mutation that constitutively activates the PI3K‐AKT pathway. However, how AKT1^E17K^ is regulated in cancer pathogenesis remains elusive. Here, RNA immunoprecipitation sequencing (RIP‐seq) is performed to interrogate the AKT1^E17K^‐interacting lncRNAs and identify that SVIL‐AS1 preferentially binds to AKT1^E17K^ rather than AKT1^WT^ proteins. It is found that SVIL‐AS1 enhances AKT1 phosphorylation and downstream signaling. SVIL‐AS1 knockdown dramatically inhibits the growth of AKT1^E17K^ cells in vitro and in vivo. Notably, AKT1 and SVIL‐AS1 interaction is AKT1 phosphorylation‐dependent. SVIL‐AS1 also interacts with PPP2R2A, a subunit of phosphatase PP2A holoenzyme, and blocks the binding of PPP2R2A to AKT1^E17K^ to prevent AKT1 dephosphorylation. Moreover, AKT1^E17K^ cells are not effectively inhibited by the allosteric AKT inhibitor, whereas silencing SVIL‐AS1 sensitizes AKT1^E17K^ cells to AKT1 allosteric inhibitor, as well as the PI3Kα inhibitor. In breast cancer tissues, SVIL‐AS1 is highly expressed and associated with p‐AKT1 level and poor prognosis of patients. Together, the findings discover a novel lncRNA regulator of mutant oncoprotein which preferentially prevents AKT1^E17K^ dephosphorylation. Targeting SVIL‐AS1 may help to improve the responses to inhibitors of the PI3K‐AKT pathway, especially in AKT1^E17K^ mutant tumors.

## Graphical abstract

Wang et al. discover a novel lncRNA regulator of mutant oncoprotein which prevents AKT1^E17K^ dephosphorylation. SVIL‐AS1 overexpression correlates with poor prognosis of breast cancer patients. It interacts with AKT1^E17K^ protein and impedes PP2A holoenzyme from dephosphorylating AKT1 by blocking the binding of the regulatory subunit PPP2R2A to AKT1, which sustains AKT1 hyperphosphorylation and strengthens AKT1^E17K^ oncogenic function.

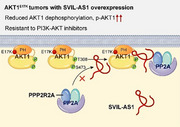

## 1 Introduction

AKT1, also known as protein kinase B (PKB), is a key node in the PI3K‐AKT pathway and regulates a variety of biological and pathological processes, such as cell proliferation, energy metabolism, and tumor progression.^[^ ^1^, ^2^ ^]^ E17K is the most common mutation of AKT1, which is in the Pleckstrin Homology (PH) domain, the lipid‐binding pocket of AKT1.^[^ ^3^ ^]^ This gain‐of‐function mutation alters the electrostatic interactions of the pocket and the lipid‐binding capacity, with increased affinity to PI(3,4,5)P3 and broadened affinity to PI(4,5)P2, thereby constitutively targeting the PH domain to the plasma membrane and leading to abnormal activation of AKT1.^[^ ^4^, ^5^ ^]^ Moreover, E17K mutation perturbates the interdomain interactions of the PH domain and the kinase domain, which disrupts the autoinhibition and results in the activation of AKT1 and downstream signaling.^[^ ^6^ ^]^

AKT1^E17K^ occurs in breast cancer, bladder cancer, endometrial cancer, colorectal cancer and acute lymphoblastic leukemia, etc.^[^ ^7^, ^8^ ^]^ In breast cancer, AKT1^E17K^ has been identified in 2% to 7% of patients and associated with poor prognosis.^[^ ^9^ ^]^ In non‐small cell lung carcinoma, AKT1^E17K^ enhances K64 methylation by SETDB1, leading to its hyper‐activation and promotes tumor development.^[^ ^10^ ^]^ However, the oncogenic potential of this mutation in different cell contexts is controversial. AKT1^E17K^ shows transforming capacity in fibroblasts in vitro and induces leukemia in vivo,^[^ ^4^ ^]^ whereas it is not sufficient for mammary epithelia cells to transform in vitro and in vivo,^[^ ^11^, ^12^ ^]^ suggesting that AKT1^E17K^ may need cooperative genetic events to achieve its full oncogenic potential.

Long non‐coding RNAs (lncRNA) are transcripts of more than 200 nucleotides which can form complex spatial structures.^[^ ^13^ ^]^ LncRNAs can bind to nucleic acids, proteins, and even lipids to exert their functions.^[^ ^13^, ^14^ ^]^ Dysregulation of lncRNA promotes the occurrence and development of a wide range of tumors.^[^ ^15^, ^16^, ^17^, ^18^, ^19^ ^]^ Protein modifications and interactions can also be regulated by lncRNAs, as we and many groups have shown.^[^ ^20^, ^21^, ^22^ ^]^ More recently, lncRNA LINK‐A has been revealed to regulate the phosphorylation of AKT1, which directly interacts with the PH domain of AKT1 and PIP3, promoting AKT1 activation in glioma and endometrial cancer.^[^ ^14^, ^23^, ^24^ ^]^ However, it remains unclear whether lncRNA can interact with mutant proteins in cancer to regulate tumor progression.

Here, we compared the lncRNAs that bind to wildtype and E17K mutant AKT1 protein and identified SVIL‐AS1 as an AKT1^E17K^‐interacting lncRNA. We found that SVIL‐AS1 could enhance the proliferation of cancer cells with AKT1^E17K^ mutation by interacting with PPP2R2A and preventing AKT1 dephosphorylation. Furthermore, we show that targeting SVIL‐AS1 can sensitize AKT1^E17K^ cancer cells to AKT1 allosteric inhibitor and the PI3Kα inhibitor.

## 2 Result

### 2.1 Identification of lncRNAs Binding to the AKT1^E17K^ Mutant Protein

**Figure 1:**
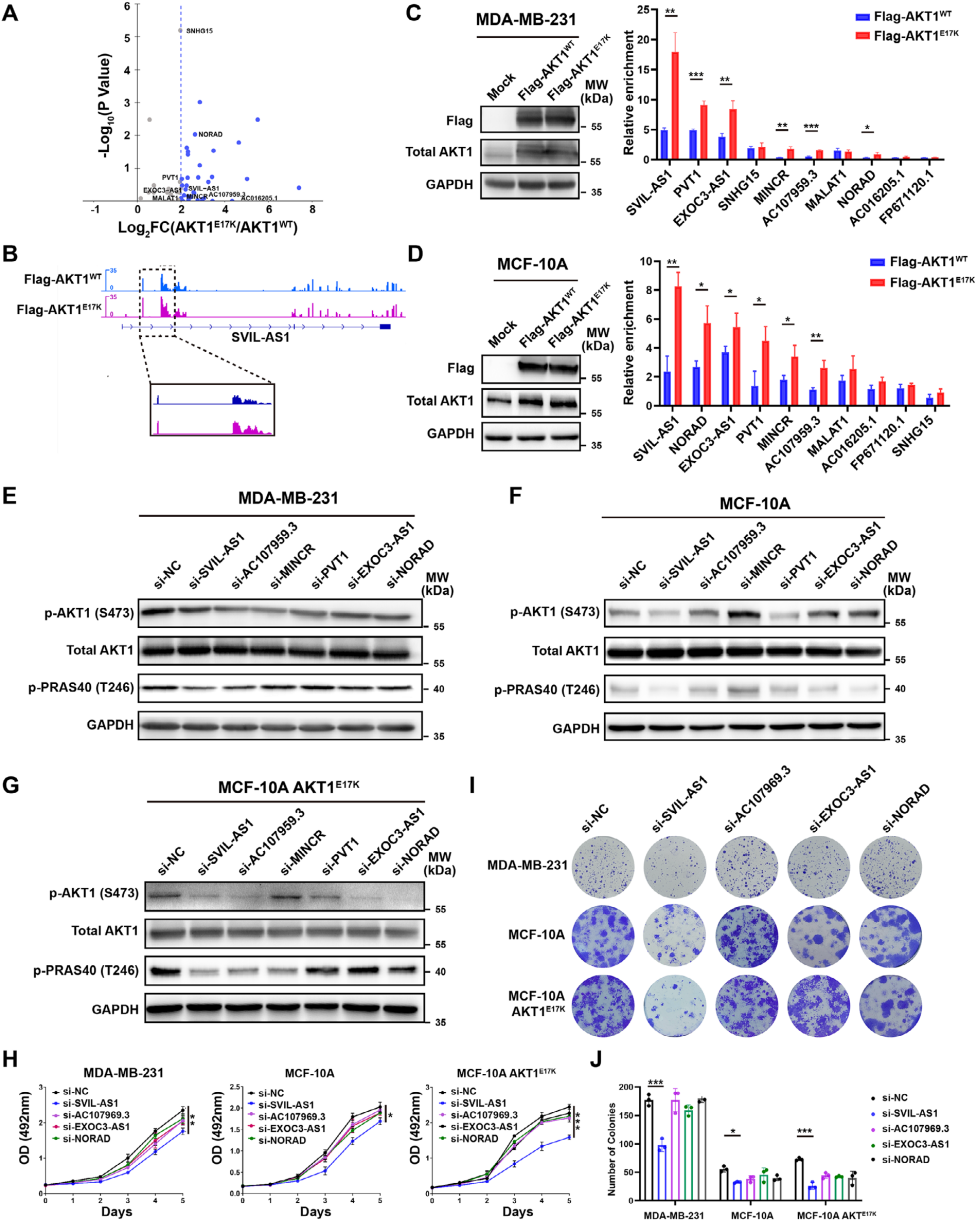
Identification of lncRNAs that bind to AKT1^E17K^ mutant protein and are essential for AKT1^E17K^ function. A) Volcano plot of RIP‐seq showing the lncRNAs with increased enrichment in the immunoprecipitates of Flag‐AKT1^E17K^ compared to Flag‐AKT1^WT^. B) The RIP‐seq tracks at the SVIL‐AS1 locus in AKT1^WT^ and AKT1^E17K^ cells. C, D) RIP and RT‐qPCR validation of top 10 lncRNAs in MDA‐MB‐231 and MCF‐10A cells. E‐G) Western blots showing the effects of silencing lncRNAs on AKT1, p‐AKT1, and p‐PRAS40 in MDA‐MB‐231, MCF‐10A, and MCF‐10A AKT1^E17K^ cells. H) Effects of silencing lncRNAs on the cell proliferation, as detected by MTS assays. I,J) Effects of silencing lncRNAs on the colony formation. The results are presented as mean ± SD of triplicate experiments. ^*^ *p* < 0.05; **p < 0.01; ***p < 0.001; ****p < 0.0001.

To identify lncRNAs binding to the AKT1**^E17K^** mutant protein, we transfected Flag‐tagged AKT1 wild‐type (AKT1^WT^) or E17K mutant (AKT1^E17K^) plasmids into breast cancer cell line MDA‐MB‐231. RNA immunoprecipitation (RIP) and deep sequencing showed that several lncRNAs preferentially bound to the AKT1**^E17K^** mutant protein (**Figure** **1**A,B). The top 10 abundant lncRNAs enriched by AKT1^E17K^ were selected for validation using RIP followed by quantitative RT‐PCR (RT‐qPCR) in MDA‐MB‐231 and MCF‐10A cells (Figure 1C,D). LncRNAs SVIL‐AS1, AC107959.3, MINCR, PVT1, EXOC3‐AS1, and NORAD were consistently enriched in the immunoprecipitates of Flag‐AKT1^E17K^, compared to Flag‐AKT1^WT^ (Figure 1C,D).

To further identify the most important lncRNA in regulating AKT1^E17K^ function, we performed western blot by silencing these lncRNAs in MDA‐MB‐231, MCF‐10A and AKT1^E17K^ knock‐in derivative cells (MCF‐10A AKT1^E17K^)^[^ ^11^ ^]^ (Figure S1A–C, Supporting Information). Knockdown of SVIL‐AS1, AC107959.3, EXOC3‐AS1, and NORAD decreased the phosphorylation levels of AKT1 and its downstream PRAS40 in all three cells (Figure 1E–G). Among these four lncRNAs, SVIL‐AS1 knockdown was the most dramatic one to suppress cell proliferation and colony formation of these three cells (Figure 1I,J). Notably, the impact of SVIL‐AS1 knockdown on AKT1 phosphorylation and cell growth was more obvious in MCF‐10A AKT1^E17K^ than in AKT1 wildtype cells (Figure 1E–J).

### 2.2 SVIL‐AS1 Binds to AKT1^E17K^ and is Essential for AKT1^E17K^ Phosphorylation

To characterize the sequence of SVIL‐AS1, we performed 5′‐ and 3′‐ rapid amplification of cDNA ends (RACE) in MCF‐10A AKT1^E17K^ cells (Figure S1D, Supporting Information). Sanger sequencing identified SVIL‐AS1 as a 1247 nt transcript which was 4 nt longer in **3**′ end than previous reported sequence (Ensemble ID: ENST00000414457) (Table S1, Supporting Information). To find out the subcellular localization of SVIL‐AS1, we extracted the nuclear and cytoplasmic RNA fractions. RT‐qPCR showed that the majority of SVIL‐AS1 was distributed in the cytoplasm of MCF‐10A and MCF‐10A AKT1^E17K^ cells (Figure S1E, Supporting Information). Fluorescence in situ hybridization (FISH) also showed the cytoplasmic localization of SVIL‐AS1 in these cells (Figure S1F, Supporting Information).

**Figure 2:**
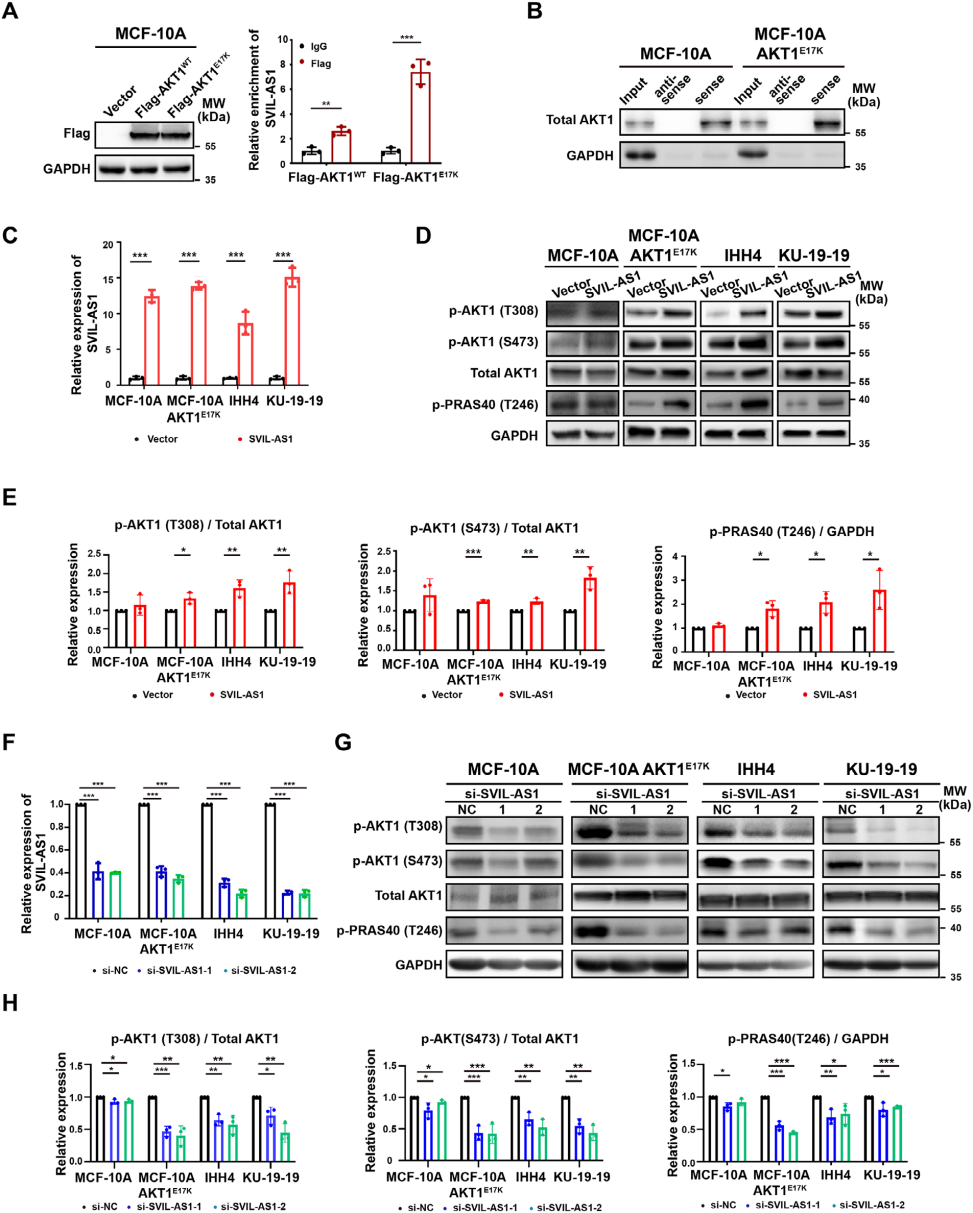
SVIL‐AS1 binds to AKT1^E17K^ and enhances AKT1 phosphorylation. A) RIP assay with the Flag antibody showing the enrichment of SVIL‐AS1 in MCF‐10A cells with Flag‐AKT1^WT^ and Flag‐AKT1^E17K^. B) RNA pulldown of SVIL‐AS1 (sense) or its antisense RNA in MCF‐10A and AKT1^E17K^ cells. Western blot was performed to detect AKT1. C) Overexpression efficiency of SVIL‐AS1 in MCF‐10A, MCF‐10A AKT1^E17K^, IHH4, and KU‐19‐19 cells, as detected by RT‐qPCR. D, E) Effects of SVIL‐AS1 overexpression on the phosphorylation of AKT1 and PRAS40. F) Knockdown efficiency of SVIL‐AS1 in MCF‐10A, MCF‐10A AKT1^E17K^, IHH4, and KU‐19‐19 cells, as detected by RT‐qPCR. G, H) Effects of SVIL‐AS1 knockdown on the phosphorylation of AKT1 and PRAS40. The results are presented as mean ± SD of triplicate experiments. *p < 0.05; **p < 0.01; ***p < 0.001; ****p < 0.0001.

To verify the interaction of SVIL‐AS1 with AKT1, we performed RIP and found that the binding capacity of SVIL‐AS1 to Flag‐AKT1^E17K^ was significantly stronger than that to Flag‐AKT1^WT^ (**Figure** **2**A). RNA pulldown also confirmed this result (Figure 2B). Then we overexpressed or silenced SVIL‐AS1 in MCF‐10A AKT1^E17K^, IHH4 (papillary thyroid carcinoma cell), and KU‐19‐19 (bladder carcinoma cell), which all had the AKT1^E17K^ mutation (Figure 2C,F). We found that SVIL‐AS1 overexpressing cells had higher phosphorylation levels of AKT1 and its downstream PRAS40 in cells with AKT1^E17K^ mutation, while the alterations were not obvious in MCF‐10A cells (Figure 2D,E). SVIL‐AS1 silenced cells had lower levels of p‐AKT1 and p‐PRAS40 in cells with AKT1^E17K^ mutation (Figure 2G,H). These results demonstrated that SVIL‐AS1 interacted with AKT1^E17K^ protein and was essential for AKT1^E17K^ phosphorylation.

### 2.3 SVIL‐AS1 Knockdown Impairs the Growth of AKT1^E17K^ Mutant Cells In Vitro and In Vivo

**Figure 3:**
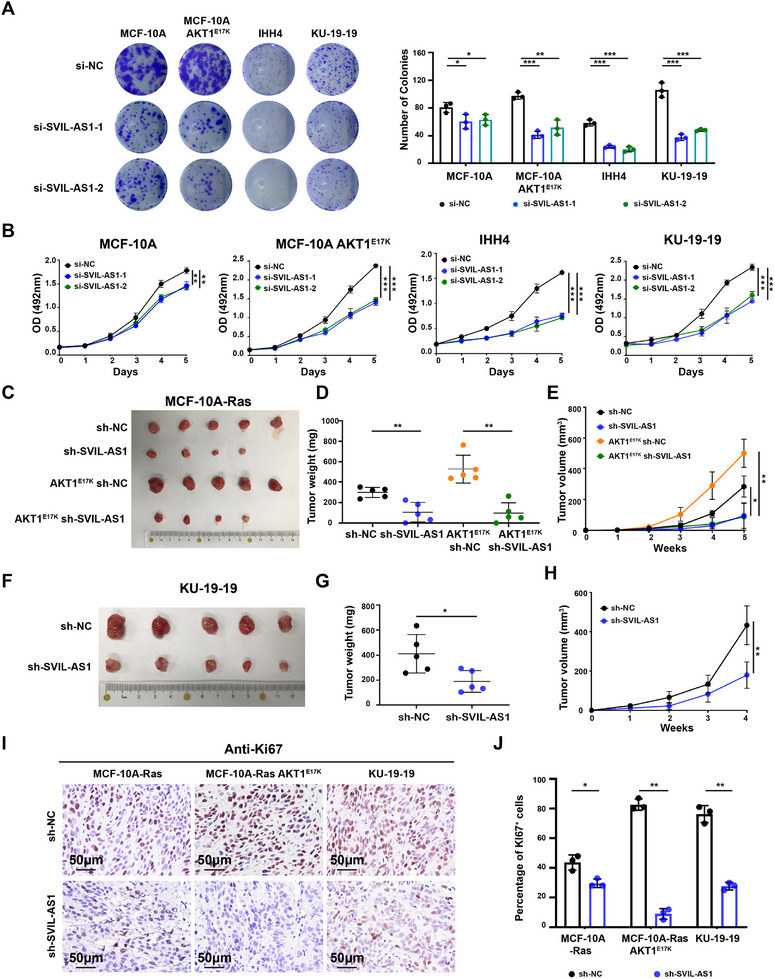
SVIL‐AS1 knockdown impairs AKT1^E17K^ cell growth in vitro and in vivo. A,B) SVIL‐AS1 knockdown reduced colony formation and cell proliferation, especially in AKT1^E17K^ mutant cell lines (MCF‐10A AKT1^E17K^, IHH4, and KU‐19‐19). C–E) Effects of knocking down SVIL‐AS1 on the tumor growth of MCF‐10A‐Ras and MCF‐10A‐Ras AKT1^E17K^ xenografts in nude mice. F–H) Effects of knocking down SVIL‐AS1 on the tumor growth of KU‐19‐19 xenografts in nude mice. I) Silencing SVIL‐AS1 decreased the percentage of Ki67‐positive cells in the xenografts. Scale bar, 50 µm. The results are presented as mean ± SD of triplicate experiments. *p < 0.05; **p < 0.01; ***p < 0.001; ****p < 0.0001.

We compared the impact of SVIL‐AS1 on the growth of wild‐type AKT1 and E17K mutant cells. Colony formation assay showed that SVIL‐AS1 knockdown reduced the clonogenicity more obviously in the MCF‐10A AKT1^E17K^ than in MCF‐10A cells (**Figure** **3**A). Similarly, silencing SVIL‐AS1 remarkably reduced the colony formation of IHH4 and KU‐19‐19 cells (Figure 3A). In parallel, MTS assays demonstrated that SVIL‐AS1 knockdown impaired the proliferation of cells with AKT1^E17K^ mutation more dramatically than wild‐type cells (Figure 3B).

To further explore the function of SVIL‐AS1 in vivo, SVIL‐AS1 knockdown cells and control cells were injected into nude mice. In line with previous reports,^[^ ^25^ ^]^ KRAS G12V‐transfected MCF‐10A AKT1^E17K^ cells formed bigger tumors than MCF‐10A cells (Figure 3C–E). The growth rate, tumor volume, and tumor weight were reduced in SVIL‐AS1 knockdown MCF‐10A AKT1^E17K^ xenografts and the reduction was more obvious in AKT1^E17K^ than in wildtype xenografts (Figure 3C–E). Knockdown of SVIL‐AS1 also decreased the tumor growth of KU‐19‐19 xenografts (Figure 3F–H). The efficiency of SVIL‐AS1 knockdown in vivo was confirmed by RT‐qPCR and ISH (Figure S2A–C, Supporting Information). The immunohistochemistry (IHC) of Ki67 also confirmed that SVIL‐AS1 knockdown reduced cell proliferation in vivo (Figure 3I,J). Moreover, IHC results showed that SVIL‐AS1 knockdown could inhibit AKT1 phosphorylation in both MCF‐10A (KRAS G12V‐transfected) and KU‐19‐19 xenografts (Figure S2D, Supporting Information). These results indicated that SVIL‐AS1 promoted cell proliferation, especially in cells with AKT1^E17K^ mutation in vitro and in vivo.

### 2.4 SVIL‐AS1 Interacts with PPP2R2A to Prevent AKT1 Dephosphorylation

**Figure 4:**
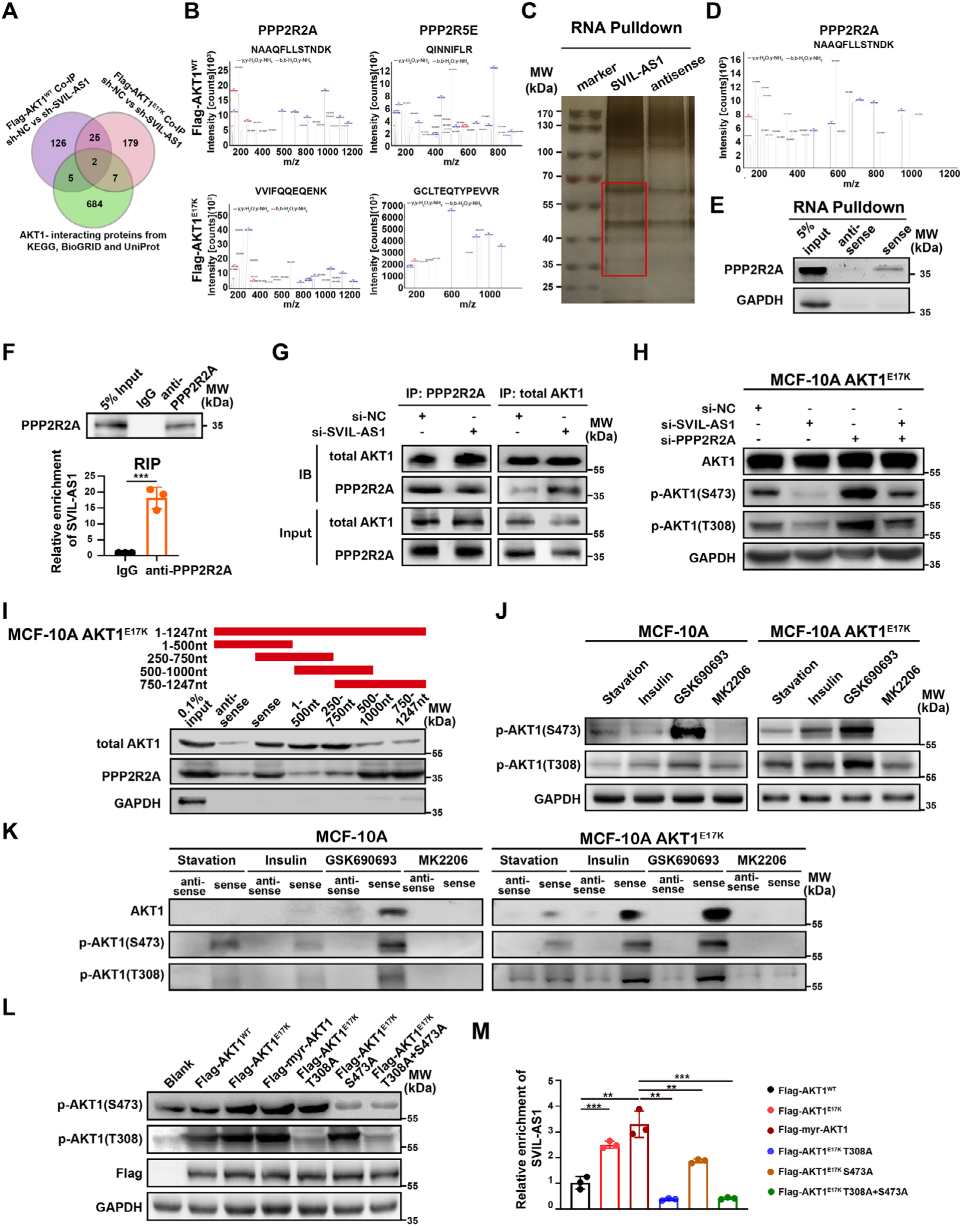
SVIL‐AS1 interacts with PPP2R2A to prevent AKT1 dephosphorylation. A) Venn diagram shows the screening of Flag‐AKT1^WT^ and Flag‐AKT1^E17K^ immunoprecipitated proteins in SVIL‐AS1 knockdown, but not in control MCF‐10A cells. Differential proteins identified by MS overlapped with AKT1‐interacting protein lists from KEGG, BioGRID, and UniProt databases. B) MS profiles of PPP2R2A and PPP2R5E in Flag‐AKT1^WT^ and Flag‐AKT1^E17K^ transfected MCF‐10A cells with SVIL‐AS1 knockdown. C) RNA pulldown using SVIL‐AS1 (sense) or the antisense RNA, followed by silver staining. Differential bands in red frames were sent for MS analysis. D) Identification of PPP2R2A by MS in SVIL‐AS1‐pulled down proteins in AKT1^E17K^ MCF‐10A cells. E) RNA pulldown of SVIL‐AS1 in MCF‐10A^E17K^ cells followed by western blot to detect PPP2R2A. F) RIP of PPP2R2A in MCF‐10A AKT1^E17K^ cells. SVIL‐AS1 was enriched by PPP2R2A IP and detected by RT‐qPCR. G) SVIL‐AS1 knockdown increased the interaction between AKT1 and PPP2R2A, as assayed by PPP2R2A IP and AKT1 IP, followed by western blot. H) Effects of SVIL‐AS1 knockdown, PPP2R2A knockdown, or SVIL‐AS1 and PPP2R2A knockdown on AKT1 phosphorylation in MCF‐10A AKT1^E17K^ cells. Western blot showing the level of AKT1 phosphorylation upon in MCF‐10A AKT1^E17K^ cells. I) RNA pulldown using SVIL‐AS1 fragments to determine the interaction region with AKT1 or PPP2R2A in MCF‐10A AKT1^E17K^ cells. J) Western blot showing the level of AKT1 phosphorylation upon treatment of serum starvation, insulin stimulation, AKT1 inhibitors GSK690639 and MK2206 in MCF‐10A and MCF‐10A AKT1^E17K^ cells. K) RNA pulldown using SVIL‐AS1 or its antisense RNA, followed by western blot in MCF‐10A and AKT1^E17K^ cells treated with serum starvation, insulin stimulation, AKT1 inhibitors GSK690639 and MK2206. L) Western blot showing the level of AKT1 phosphorylation in MCF‐10A cells transfected with different forms of Flag‐AKT1. M) RIP assay with the Flag antibody showing that SVIL‐AS1 enrichment was AKT1 phosphorylation‐dependent in MCF‐10A transfected with different forms of Flag‐AKT1. The results are presented as mean ± SD of triplicate experiments. *p < 0.05; **p < 0.01; ***p < 0.001; ****p < 0.0001.

To investigate how SVIL‐AS1 specifically affected the function of AKT1^E17K^ mutant, we compared the proteins immunoprecipitated by Flag‐AKT1^WT^ and Flag‐AKT1^E17K^ in control and SVIL‐AS1 knockdown MCF‐10A cells (**Figure** **4**A). We sent the differential protein bands for mass spectrometry (MS) analysis and compared the MS‐identified proteins with the AKT1‐interacting protein lists from KEGG, BioGRID, and UniProt databases (Figure 4A; Tables S2, S3, Supporting Information). Notably, two proteins, PPP2R2A and PPP2R5E, were immunoprecipitated by Flag in SVIL‐AS1 knockdown cells but not in control AKT1^E17K^ and AKT1^WT^ cells (Figure 4A,B), suggesting that SVIL‐AS1 prevent the interaction between AKT1 and these phosphatase proteins. Next, we performed RNA pulldown using SVIL‐AS1 and its antisense RNA as a negative control. The protein band (30–70 kDa) enriched in the SVIL‐AS1 pulldown complex in MCF‐10A AKT1^E17K^ cells was cut for MS identification (Figure 4C). PPP2R2A was again characterized in SVIL‐AS1 pulldown complex in AKT1^E17K^ MCF‐10A cells (Figure 4D; Table S4, Supporting Information).

PP2A phosphatase is a heterotrimer protein complex composed of a catalytic subunit (PP2A Ca/b), a scaffold subunit (PP2A Aa/b), and a regulatory subunit (PP2A B). PPP2R2A belongs to the regulatory subunit (PP2A B). It has been reported that PP2A is a major phosphatase of AKT1.^[^ ^26^, ^27^ ^]^ To verify whether SVIL‐AS1 also interact with PP2A subunit, we performed RNA pulldown and RIP in MCF‐10A AKT1^E17K^ cells. Both these experiments confirmed the interaction of SVIL‐AS1 with PPP2R2A (Figure 4E‐F). We then performed Co‐IP in MCF‐10A AKT1^E17K^ cells using PPP2R2A antibody and found that more AKT1 bound to PPP2R2A after SVIL‐AS1 knockdown (Figure 4G). Reciprocal immunoprecipitation using AKT1 antibody confirmed this result (Figure 4G). Moreover, PPP2R2A knockdown could reverse the reduction of AKT phosphorylation caused by SVIL‐AS1 knockdown in MCF‐10A AKT1^E17K^ cells (Figure 4H). These results indicated that SVIL‐AS1 specifically affected the phosphorylation level of AKT1^E17K^ mutant via PPP2R2A.

To further determine the interaction region of SVIL‐AS to AKT1 and PPP2R2A, we generated a series of SVIL‐AS1 fragments and performed RNA pulldown. The 1–500 and 250–750 nt fragment could bind to AKT1, and 500–1000 and 750–1247 nt fragment could bind to the PPP2R2A (Figure 4I), which indicated that SVIL‐AS1 250–500 nt was the AKT1^E17K^‐interaction region and 750–1000 nt was the PPP2R2A‐interaction region. The above results demonstrated that SVIL‐AS1 specifically bound to PPP2R2A and prevents the interaction of AKT1^E17K^ with PPP2R2A.

### 2.5 AKT1 and SVIL‐AS1 Interaction is AKT1 Phosphorylation‐Dependent

Previous studies demonstrated that AKT1^E17K^ mutation could improve the affinity of AKT1 to cell membrane which made it easily to be phosphorylated.^[^ ^5^ ^]^ We altered AKT1 phosphorylation levels in MCF‐10A and MCF‐10A AKT1^E17K^ cells by serum starvation, insulin stimulation, and treatment of AKT1 inhibitor, and then explored the interaction of SVIL‐AS1 with AKT1 and AKT1^E17K^ proteins. As expected, AKT1 phosphorylation level increased upon insulin stimulation and the treatment of the ATP‐competitive inhibitor GSK690693, while the AKT1 phosphorylation level decreased upon serum starvation and the treatment of AKT1 allosteric inhibitor MK2206 (Figure 4J). Then we performed RNA pulldown and found that the extent of SVIL‐AS1 interaction with AKT1 was in line with the level of AKT1 phosphorylation (Figure 4K), suggesting that this interaction was AKT1 phosphorylation‐dependent.

To further determine which phosphorylation site was critical for the interaction of SVIL‐AS1 with AKT1, we transfected Flag‐tagged AKT1 T308A, S473A, and T308A+S473A constructs into MCF‐10A cells. The constitutively active myristoylated AKT1 (myr‐AKT1) was used as a positive control (Figure 4L). RIP experiments using the Flag antibody showed that SVIL‐AS1 was enriched most in the myr‐AKT1 transfected cells, while T308 mutation could obviously impair the interaction of AKT1 and SVIL‐AS1 (Figure 4M). This result indicated that T308 phosphorylation was important for the interaction of AKT1 and SVIL‐AS1.

### 2.6 Targeting SVIL‐AS1 Sensitizes AKT1^E17K^ Cells to AKT1 Allosteric Inhibitor and PI3Kα Inhibitor

**Figure 5:**
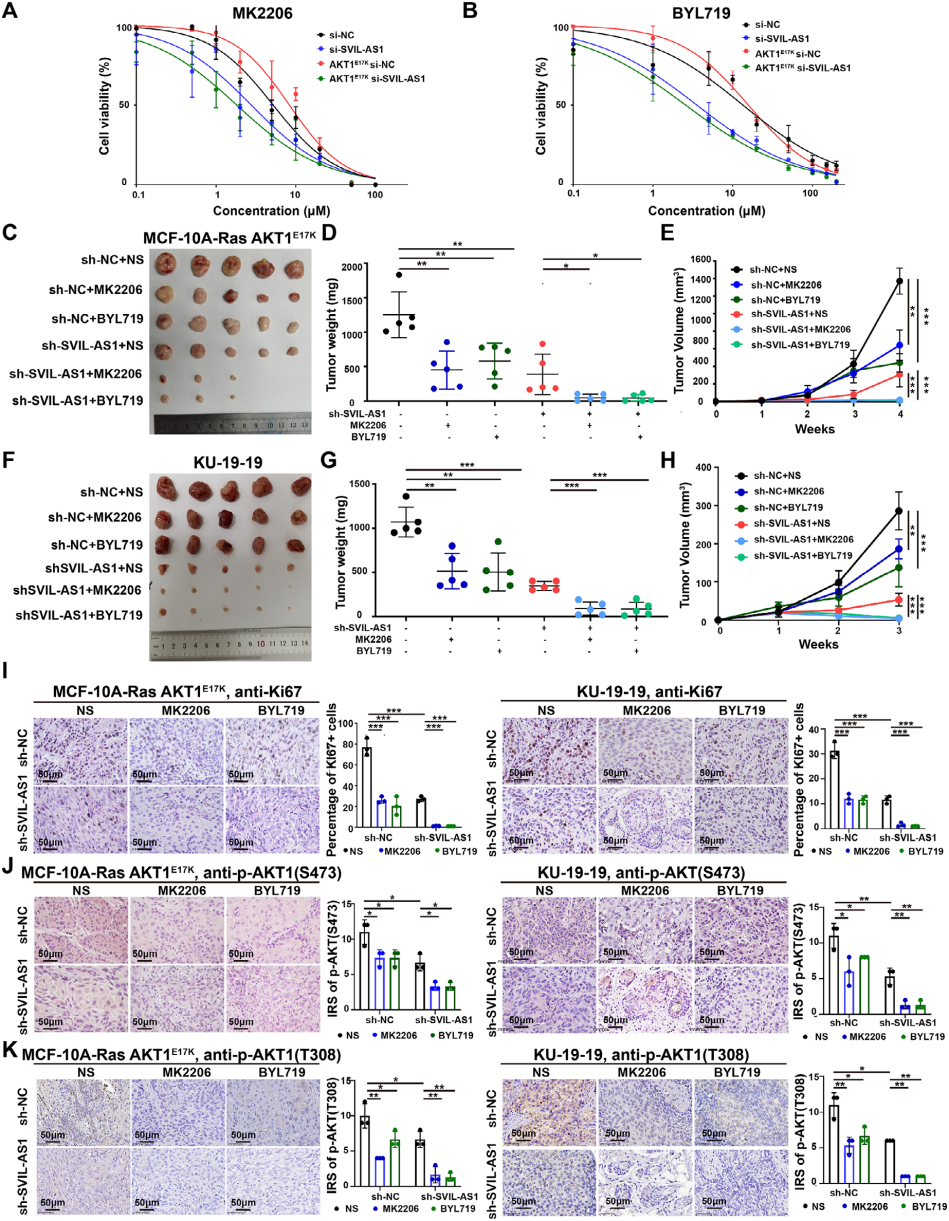
Targeting SVIL‐AS1 sensitizes AKT1^E17K^ cells to AKT1 allosteric inhibitor and PI3Kα inhibitor. A) SVIL‐AS1 knockdown sensitized MCF‐10A cells to AKT1 allosteric inhibitor MK2206, especially E17K mutant cells. B) SVIL‐AS1 knockdown sensitized MCF‐10A cells to PI3Kα inhibitor BYL719, especially E17K mutant cells. C–H) Effects of SVIL‐AS1 knockdown together with BYL719 or MK2206 on regulating tumor growth of MCF‐10A AKT1^E17K^ and KU‐19‐19 xenografts in nude mice. I–K) SVIL‐AS1 knockdown decreased the percentage of Ki67, p‐AKT1 (S473), and p‐AKT1 (T308) positive cells in the xenografts. Scale bar, 50 µm. The results are presented as mean ± SD of triplicate experiments. *p < 0.05; **p < 0.01; ***p < 0.001; ****p < 0.0001.

Preclinical evidence suggests that cancers with the AKT1^E17K^ mutation are more resistant to AKT1 allosteric inhibitors.^[^ ^28^ ^]^ To detect whether SVIL‐AS1 affected the response to AKT1 allosteric inhibitor, we performed MTS assays and found that SVIL‐AS1 knockdown sensitized both MCF‐10A and AKT1^E17K^ cells to AKT1 allosteric inhibitor MK2206, and the effect was more dramatic in AKT1^E17K^ cells (**Figure** **5**A). Moreover, SVIL‐AS1 knockdown could also sensitize cells to PI3Kα inhibitor BYL719, especially the AKT1^E17K^ cells (Figure 5B).

Similar to the results in vitro, combining SVIL‐AS1 knockdown and MK2206 or BYL719 therapy induced a significant reduction in tumor growth compared with that of MK2206 or BYL719 alone in MCF‐10A‐Ras AKT1^E17K^ and KU‐19‐19 xenografts (Figure 5C–H). Immunostaining in these xenografts demonstrated that the percentage of Ki67^+^ cells in SVIL‐AS1 knockdown combining with MK2206 or BYL719 therapy decreased obviously comparing to SVIL‐AS1 knockdown (Figure 5I). The level of p‐AKT1 (S473 and T308) also reduced in SVIL‐AS1 knockdown combining with MK2206 or BYL719 therapy (Figure 5J,K). Therefore, targeting SVIL‐AS1 could further sensitize tumors with AKT1^E17K^ mutation to AKT1 allosteric inhibitor and PI3Kα inhibitor.

### 2.7 SVIL‐AS1 is Highly Expressed in Breast Cancer and Associated with p‐AKT1 Levels and the Poor Prognosis of Patients

**Figure 6:**
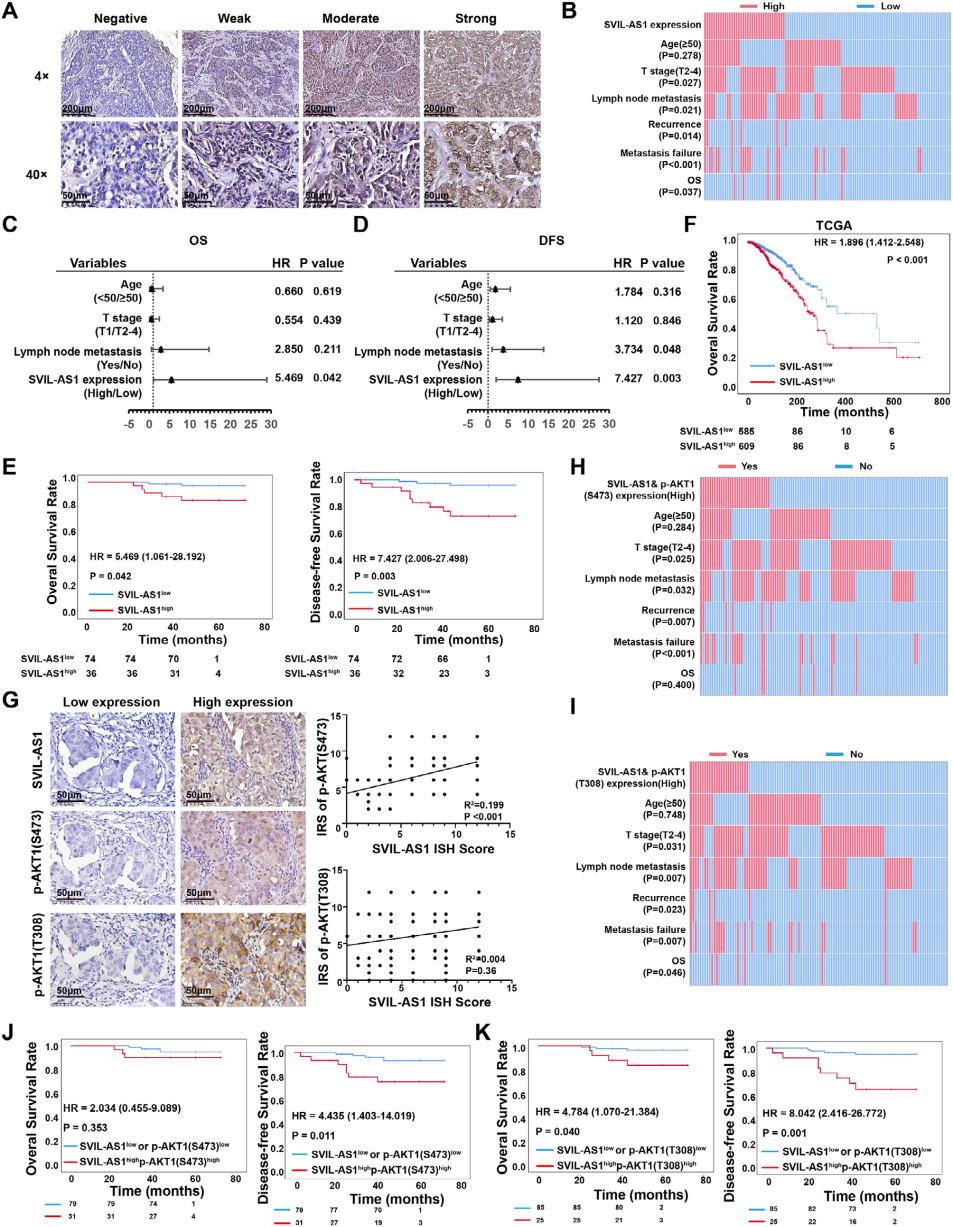
SVIL‐AS1 is highly expressed in breast cancer and is associated with poor prognosis. A) Representative ISH results of SVIL‐AS1 expression in breast cancer tissues. Positive ISH signals are detected as brown staining, and nuclei are counterstained with hematoxylin. B) Correlations between the expression of SVIL‐AS1 and the clinicopathologic features of breast tumors. C,D) Univariate analyses analysis of OS and DFS in breast cancer patients. E) Kaplan–Meier survival curves showing high SVIL‐AS1 expression correlate with worse OS and DFS in our breast cancer cohort. F) Kaplan‐Meier survival analysis of SVIL‐AS1 in TCGA breast cancer database. G) The association of SVIL‐AS1 expression with p‐AKT1 (S473) or p‐AKT1 (T308) in breast tumors. H,I) Correlation between SVIL‐AS1^high^ p‐AKT1(S473)^high^ or SVIL‐AS1^high^ p‐AKT1(T308) ^high^ with the clinicopathologic features of breast tumors. J,K) Kaplan‐Meier survival curves showing SVIL‐AS1^high^ p‐AKT1 (S473)^high^ or SVIL‐AS1^high^ p‐AKT1 (T308) ^high^ correlates with worse OS and DFS in our breast cancer cohort.

We next performed ISH in breast cancer tissues to assess the association of SVIL‐AS1 with clinical and pathological parameters. SVIL‐AS1 was detected in 95.45% (105/110) of breast cancer tissues, which are mainly in the cytoplasm of breast cancer cells (**Figure** **6**A). SVIL‐AS1 expression was significantly associated with T stage (*p* = 0.027), N stage (*p* = 0.021), recurrence (*p* = 0.014), and metastasis failure (*p* < 0.001) of breast cancer patients (Figure 6B). Univariate Cox regression analysis showed that SVIL‐AS1 expression was a prognostic factor for overall survival (OS) (*p* = 0.042) and disease‐free survival (DFS) (*p* = 0.003) in breast cancer patients (Figure 6C,D). Similar results were obtained by Kaplan‐Meier analysis in our cohort (Figure 6E). In the TCGA breast cancer database, the cumulative 5‐year OS and 10‐year OS were 93.2% and 80.4% in the SVIL‐AS1^high^ group, whereas 96.6% and 90.2% in the SVIL‐AS1^low^ group (Figure 6F). Kaplan‐Meier analysis also demonstrated that high level of SVIL‐AS1 significantly correlated with reduced OS of breast cancer patients in TCGA dataset (Figure 6F). Moreover, we analyzed SVIL‐AS1 expression in breast cancer subtypes and other cancer types. There was no significant difference between luminal and HER2+ subtypes, but triple‐negative breast tumors showed higher SVIL‐AS1 expression (Figure S3A,B, Supporting Information). In TCGA dataset, overexpression of SVIL‐AS1 was observed in head and neck squamous carcinoma and hepatocellular carcinoma when compared to normal tissues (Figure S3C, Supporting Information).

Further, we detected AKT1 phosphorylation in breast cancer tissues by immunostaining. A positive correlation was found between the expression of SVIL‐AS1 and the level of p‐AKT1(S473) (*p* < 0.001) (Figure 6G). Both SVIL‐AS1^high^p‐AKT1(S473)^high^ and SVIL‐AS1^high^p‐AKT1(T308)^high^ were significantly associated with T stage, N stage, recurrence, and metastasis failure of breast cancer patients (Figure 6H–I). SVIL‐AS1^high^p‐AKT1(S473)^high^ and SVIL‐AS1^high^ p‐AKT1(T308)^high^ were also associated with shorter DFS time (Figure 6J,K). However, the combination of SVIL‐AS1 and p‐AKT1 did not predict the outcomes of breast cancer patients better than SVIL‐AS1 alone, indicating that SVIL‐AS1 is a potent biomarker of patient outcome.

## 3 Discussion

**Figure 7:**
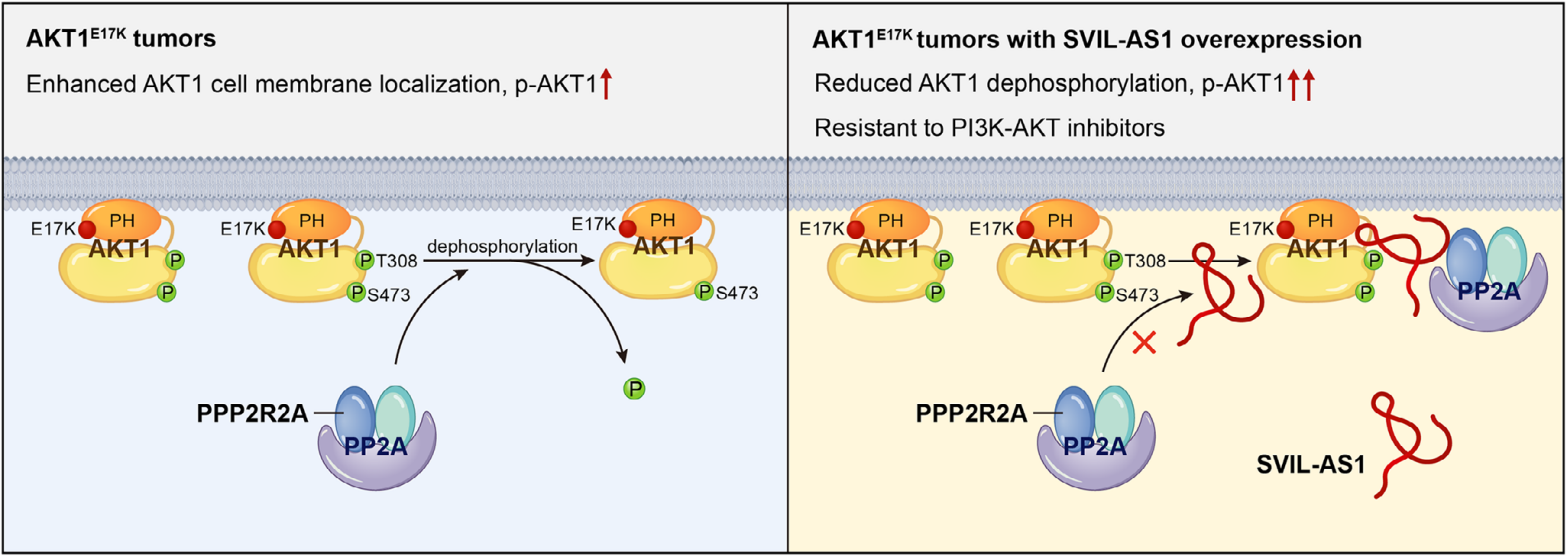
Graphic working model of SVIL‐AS1. LncRNA SVIL‐AS1 interacts with AKT1^E17K^ mutant protein, which prevents PP2A holoenzyme from dephosphorylating AKT1 by blocking the binding of the regulatory subunit PPP2R2A to AKT1, thus sustaining AKT1 hyperphosphorylation and strengthening AKT1^E17K^ oncogenic function.

AKT1^E17K^ mutation can enhance the affinity of AKT1 to cell membrane, making it easily to be phosphorylated.^[^ ^4^, ^5^ ^]^ Previous studies focused on the oncogenic mechanism of AKT1^E17K^ of increasing membrane localization and promoting phosphorylation. However, whether the phosphorylation status of AKT1^E17K^ is regulated by other factors is largely unknown. In this study, we discover that lncRNA SVIL‐AS1 specifically interacts with AKT1^E17K^ mutant, which prevents PP2A holoenzyme from dephosphorylating AKT1^E17K^ by blocking the binding of PPP2R2A to AKT1^E17K^ (**Figure** **7**). To our knowledge, this is the first study demonstrating the dephosphorylation of AKT1^E17K^ is regulated by a lncRNA.

Only a small number of lncRNAs have been functionally characterized in the human genome. The role of SVIL‐AS1 in biological and pathological processes is little known. We identify SVIL‐AS1 from AKT1 RIP‐seq and validate it as an AKT1^E17K^‐interacting lncRNA using RIP‐qPCR and RNA pulldown. Moreover, we uncover that SVIL‐AS1 enhances AKT1^E17K^ phosphorylation and downstream signaling, thus promoting the proliferation of AKT1^E17K^ cells in vitro and in vivo. These data elucidate the critical role of SVIL‐AS1 in sustaining AKT1^E17K^ activation and facilitating tumor growth. Of note, we reveal that SVIL‐AS1 can also bind to wild‐type AKT1. However, the function of SVIL‐AS1 is dependent on AKT1 phosphorylation. Thus, SVIL‐AS1 shows higher binding capacity and oncogenic activity in AKT1^E17K^ cells than in AKT1^WT^ cells.

Phosphorylation at both T308 and S473 is required for full Akt activity.^[^ ^2^ ^]^ Dephosphorylation of AKT1 at Thr308 is mainly mediated by PP2A.^[^ ^26^, ^27^ ^]^ Conformational change induced by dephosphorylation at T308 can expose S473 to phosphatases, thereby making it more susceptible to dephosphorylation as well.^[^ ^29^ ^]^ Thus, dephosphorylation of AKT1 by PP2A will lead to AKT1 inactivation. Access of PP2A to AKT1 can be restricted by proteins such as BTBD10,^[^ ^30^ ^]^ as well as AKT1 conformational change and intramolecular interactions.^[^ ^31^, ^32^ ^]^ Here, our findings elucidate a previously unrecognized mechanism of lncRNA SVIL‐AS1 in blocking the access of PP2A to phosphorylated AKT1, which prevents AKT1 dephosphorylation. LncRNAs have been demonstrated to bind to proteins and modulate the protein post‐translational modifications, interactions, and activities.^[^ ^33^ ^]^ Unlike the regulation at protein levels, RNA biosynthesis costs much less nutrients and ATP.^[^ ^34^ ^]^ If lncRNAs can comply the same function as proteins to regulate post‐translational modifications, it will be a more economical mechanism to regulate the cell signaling. Fast‐growing cancer cells may take advantage of this mechanism and use more lncRNAs to fulfill such functions, thus optimizing the usage of cellular resources, although this notion still needs to be further validated and tested in different organisms.

The components of the PI3K‐AKT pathway are important targets for drug development. Previous study showed that AKT1^E17K^ mutants were not effectively inhibited by allosteric AKT inhibitors.^[^ ^4^, ^6^ ^]^ In our study, we found that SVIL‐AS1 knockdown could decrease the IC50 of MK2206 more obviously in MCF‐10A AKT1^E17K^ cells and significantly inhibited the proliferation of AKT1^E17K^ mutant subcutaneous tumors. We also tested the effects of knocking down SVIL‐AS1 in combination with the most frequently used PI3K inhibitor BYL719 (Alpelisib). Knockdown of SVIL‐AS1 could also increase the sensitivity of AKT1^E17K^ mutant cells to BYL719. These data suggest that SVIL‐AS1 may serve as a novel therapeutic target toward tumors with AKT1^E17K^ mutation and reverse the resistance to allosteric AKT inhibitors and PI3K inhibitors. Recent translation studies have shown that lncRNAs can be knocked down by RNAi, antisense oligonucleotides (ASOs), CRISPR‐based approaches, and small molecules that target RNA‐protein interactions.^[^ ^35^ ^]^ In the future, technological advancements may enable SVIL‐AS1 targeting in combination with PI3K‐AKT1 inhibitors for cancer therapeutics.

In this study, breast cancer tissues were not categorized by AKT1 mutation status. Given the relatively low prevalence of AKT1 mutations in breast cancer, the majority of specimens are AKT1^WT^ tumors. Even so, high level of SVIL‐AS1 significantly correlated with reduced survival of breast cancer patients in both TCGA dataset and our cohort, and SVIL‐AS1 expression correlates with p‐AKT1 (S473) in breast cancer tissues. This may be because SVIL‐AS1 can also bind to AKT1^WT^ protein when AKT1 is phosphorylated, and SVIL‐AS1 knockdown can reduce cell growth in AKT1^WT^ tumors, although the effect is not as dramatic as in AKT1^E17K^ tumors. Nevertheless, it remains crucial to distinguish AKT1^WT^ and AKT1^E17K^ status in further translational and clinical studies when targeting SVIL‐AS1 in combination with PI3K‐AKT1 inhibitors for more precise evaluation of treatment responses of cancer patients.

Together, our work discovered a novel lncRNA regulator of AKT1 dephosphorylation, which preferentially acts on the AKT1^E17K^ mutant to strengthen its oncogenic function. The development of strategies targeting SVIL‐AS1 may help to improve the responses to inhibitors of the PI3K‐AKT pathway, especially in AKT1^E17K^ mutant tumors.

## 4 Experimental Section

### Patients and Tissue Samples

In our cohort, 110 breast cancer samples were collected from patients diagnosed between 2016 and 2018 in Sun Yat‐sen Memorial Hospital, Sun Yat‐sen University. The median follow‐up time was 48 months (21–72 months). All tumor samples were collected from patients with informed consent, and approved by the Institute Research Ethics Committee of Sun Yat‐sen Memorial Hospital, Sun Yat‐sen University (SYSKY‐2023‐384‐01).

### Cell Cultures

MCF‐10A, MDA‐MB‐231, KU‐19‐19, IHH4, and HEK293FT were obtained from the American Type Culture Collection (ATCC) and were cultured according to standard protocols. MCF‐10A were cultured in DMEM/F12(Gibco, USA) supplemented with EGF (peprotech, USA), hydrocortisone (Sigma, USA), cholera toxin (Sigma, USA), insulin (Sigma, USA) and 5% horse serum (Invitrogen, USA). IHH4, HEK293FT, and MDA‐MB‐231 cell lines were cultured in DMEM (Gibco, USA) with 10% fetal bovine serum (Gibco, USA). KU‐19‐19 was cultured in RPMI1640 (Gibco, USA) with 10% fetal bovine serum (Gibco, USA). All the cell lines were cultured at 37 °C with 5% CO_2_. The cells were free from mycoplasma contamination.

### RNA Immunoprecipitation (RIP)

Cell lysates were collected with IP lysis buffer supplementary with protease inhibitors, phosphatase inhibitors, RNase inhibitors, and EDTA. The cells were lysed at 4 °C for 20 min and centrifuged at 4 °C at 12 000 g for 20 min. The supernatant was transferred to a new EP tube. 30ul were reserved for transfection efficiency testing, and 5% of the volume was taken as input storing at −80 °C. The remaining supernatant was divided equally and incubated separately with magnetic beads coupled with specific protein antibodies or control IgG. The incubation was carried out overnight at 4 °C with rotation. The magnetic rack was used for collecting magnetic beads. The beads were washed by 500ul of TBST for 3–5 times. RNA was extracted by TRIzol reagent and was used for sequencing or PCR.

### Fluorescence In Situ Hybridization (FISH) and ISH

The probe of 5′digoxin‐labeled SVIL‐AS1 was TGCAGAGTAGGACTGACATAA. FISH was performed by fluorescent in situ hybridization kit (C10910, RIBOBIO, China) according to the manufacturer's instructions. Confocal images were acquired using a confocal laser scanning microscope (Zeiss LSM710, Germany). ISH was performed on paraffin‐embedded sections with in situ hybridization detection kit I (POD, Boster Biological Technology, Wuhan, China). Briefly, after dewaxing and rehydration, the samples were digested with proteinase K and hybridized with the 5′digoxin‐labeled probe at 55 °C overnight. The sections were then incubated overnight at 4 °C with anti‐digoxin monoclonal antibody (Roche, Basel, Switzerland) and stained with DAB (3,3′‐diaminobenzidine) and hematoxylin.

### Rapid Amplification of cDNA Ends (RACE)

RACE assay was performed using HiScript‐TS 5′/3′ RACE Kit (634 923, Clontech, Mountain View, CA) according to the manufacturer's instructions. The primers were used as follows: (3′ RACE: 5′‐ GATTACGCCAAGCTTTGCAGCGGGGTTAGGATGGACG AGG‐3′, 5′RACE: 5′‐ GATTACGCCAAGCTTCTCTGGGGCTGGGCTGAGCAAATG C‐3′).

### RNA Pulldown

The biotin‐labeled SVIL‐AS1 was transcribed with MEGAscript T7 in vitro transcription kit (AMB13345, Invitrogen, USA) according to the manufacturer's instructions. To allow proper secondary structure formation, 5 pmol of biotinylated RNA in RNA structure buffer was heated to 95 °C for 2 min, then put on ice for 3 min, and left at room temperature for 30 min. The cell lysates were incubated with folded RNA in RIP buffer at 4 °C overnight. The Dynabeads Streptavidin magnetic beads (M280, Invitrogen, USA) were added to each binding reaction and incubated at RT for 1h. After being washed by RIP buffer for 5 times, the samples were then collected for mass spectrometry analysis or western blot.

### Animal Experiments

All the in vivo experiments were approved and supervised by the Animal Ethics Committee of Sun Yat‐sen Memorial Hospital, Sun Yat‐sen University (AP20220152). Female BALB/c nude mice (4‐5 weeks old) were purchased from Gempharmatech (Guangdong). All the mice were raised under standard conditions (20–26 °C temperature, 40–60% humidity) with a 12 h light/12 h dark cycle at the specific‐pathogen‐free (SPF) animal facility in the Laboratory Animal Resource Center of Sun Yat‐Sen University.

MCF‐10A (KRAS G12 V) sh‐NC or sh‐SVIL‐AS1 cells, MCF‐10A AKT1^E17K^ (KRAS G12 V) sh‐NC or sh‐SVIL‐AS1 cells were injected into the mammary fat pads of nude mice at a dose of 2 × 10^6^ 100 µL^−1^ containing 20% Matrigel. KU‐19‐19 sh‐NC cells and KU‐19‐19 sh‐SVIL‐AS1 cells were subcutaneously inoculated into nude mice at a dose of 2 × 10^6^ 100µL^−1^ containing 20% Matrigel. To induce Tet‐on shRNA expression, mice were fed with doxycycline (2000 ppm) via diet.

In the treatment experiments using MK2206 or BYL719, MCF‐10A‐Ras of sh‐NC or sh‐SVIL‐AS1 cells, KU‐19‐19 sh‐NC or sh‐SVIL‐AS1 cells were all subcutaneously inoculated into nude mice at a dose of 5 × 10^6^ 100µL^−1^ containing 20% Matrigel. To induce Tet‐on shRNA expression, mice were fed with doxycycline (2000 ppm) via diet. The mice were given BYL719 (25mg kg^−1^, q3d), MK‐2206 (240mg kg^−1^, q3d) or normal saline by intragastric injection. The tumor volumes (mm^3^) were calculated according to the formula width^2^ × length.

### Statistical Analysis

The statistical analysis was performed using GraphPad Prism. The association of SVIL‐AS1 with clinicopathological features was analyzed by either χ2 test or Fisher's exact test. 2‐tailed Student's *t*‐test was used to analyze differences between two groups of variables. Univariate regression analysis was performed using the Cox proportional hazard model. Survival curves were performed by Kaplan‐Meier survival analysis and the log‐rank test. *p*‐value < 0.05 was considered statistically significant.

## Conflict of Interest

The authors declare no conflict of interest.

## Author Contributions

J.W. and W.C. contributed equally to this work. M.L.L. and H.H. conceived ideas and designed the study; M.L.L. and X.L. supervised the study; J.W. and W.C. performed most of the experiments and analysis; J.W., R.Y., and Q.L. performed in vivo study; P.H. and X.H. collected the patients’ information; J.W. drafted the manuscript; M.L.L. edited the manuscript. All authors reviewed the manuscript.

## Supporting information



Supporting Information

## Data Availability

The RIP‐seq data have been deposited in GEO and are publicly available on the date of publication. Accession number is GSE268685 with the secure token szwxqsomxxgjrwn for review.
