# Supplementary material for: AKT1E17K‐Interacting lncRNA SVIL‐AS1 Promotes AKT1 Oncogenic Functions by Preferentially Blocking AKT1E17K Dephosphorylation
Source: Adv Sci (Weinh). 2025 Mar 26;12(19):2500919. doi: 10.1002/advs.202500919 (PMC12097031; doi:10.1002/advs.202500919)
Supplement: Supplementary file 1 — Supporting Information [file ADVS-12-2500919-s001.pdf]

ADVANCED SCIENCE
Open Access

Supporting Information

for *Adv. Sci.*, DOI 10.1002/advs.202500919

AKT1^{E17K}-Interacting lncRNA SVIL-AS1 Promotes AKT1 Oncogenic Functions by Preferentially Blocking AKT1^{E17K} Dephosphorylation

*Jingyi Wang, Wenying Chen, Qianying Li, Ruiyi Yang, Xiaorong Lin, Ping Han, Xiaoming Huang, Hai Hu and Man-Li Luo**

Supporting Information

$AKT1^{E17K}$-interacting lncRNA SVIL-AS1 promotes AKT1 oncogenic functions by preferentially blocking $AKT1^{E17K}$ dephosphorylation

Jingyi Wang[#], Wenying Chen[#], Qianying Li, Ruiyi Yang, Xiaorong Lin, Ping Han, Xiaoming Huang, Hai Hu, Man-Li Luo[*]

Supporting Information include Supplementary Methods, Figure S1-S3, Table S1-S6.

Supplementary Methods

Constructs, cell transfection and infection: The siRNA/shRNA sequences of SVIL-AS1 (RIBOBIO) are listed in Table S5, Supporting Information. ShRNA sequences were cloned into pLKO-Tet-On plasmid for lentivirus-mediated knockdown. Full-length and truncated SVIL-AS1 constructs as well as Flag-tagged $AKT1^{WT}$, $AKT1^{T308A}$, $AKT1^{S473A}$, $AKT1^{T308A+S473A}$, myristoylated AKT1 (myr-AKT1) were cloned into pcDNA3.1 plasmids for ectopic expression. Cells were transfected with indicated siRNAs using Lipofectamine RNAiMAX (#13778150, Invitrogen), or plasmids using Lipofectamine 3000 (#L3000008, Invitrogen). After transducing with lentiviral particles, the cells were selected with puromycin (1 μg/ml) to generate stable cell lines and treated with doxycycline (100 ng/ml) to induce Tet-On shRNA expression for SVIL-AS1 knockdown.

RT-qPCR: Total RNA were extract with TRIzol reagent (Invitrogen, Carlsbad, CA). Total RNA was transcribed into cDNA with PrimeScript RT Master Mix kit (TAKARA,

RR036A). Real-time PCR was performed with ChamQ SYBR qPCR Master Mix according to manufacturer's instructions (Vazyme, Cat# Q311-02/03). The primers in the study were shown in Table S6, Supporting Information.

Western blot: Adherent cells were washed three times with PBS and were lysed by RIPA lysis buffer (CWBIO, China) containing protease inhibitors (CWBIO, China) on ice for 30 min. BCA kit (Thermo Fisher, America) was used for protein concentration determination. Primary antibodies in this study including anti-pan-AKT1 (1:1000, 4691S, CST), anti-AKT1-S473 (1:1000, 4060S, CST), anti-AKT-T308 (1:1000, 13038, CST), anti-PRAS40-T246 (1:1000, 13175, CST), anti-PPP2R2A (1:1000, 5689, CST), anti-GAPDH (1:5000, 60004-1-Ig, Proteintech). The secondary antibodies including goat anti-rabbit IgG H&L (HRP) (1:5000, ab6721, Abcam) and goat anti-mouse IgG H&L (HRP) (1:5000, ab6789, Abcam). Images were captured by photo system (Mini Chemi 910, SinSage, China).

MTS assay: The cells were pretreated to meet different experimental requirements. Depending on the growth rate of the cells, 1000-3000 cells were seeded into a 96-well plate. The culture medium was removed from the wells and added 120 microliters (20ul MTS + culture medium) MTS working solution. The mixture was incubated in the dark for 2 hours in a cell culture incubator. The absorbance was measured at 492nm at different time points. For the determination of IC50 values of drugs on cells, the OD ratio was measured 24 hours after drug addiction. 3 replicate wells were included in each analysis and at least three independent experiments were conducted.

Immunohistochemistry (IHC): The immunohistochemistry was performed using antibodies of anti-p-AKT1 (S473) (1:100, CST4060s, Cell Signaling Technology), anti-p-AKT1(T308) (1:100, ab16667, Abcam). The images were acquired by a whole slide scanner (KFBIO, China) or microscope (NIKON ECLIPSE NI). The staining intensity was scored from 0 to 3. The staining extent was scored 1 (< 25%), 2 (25%-50%), 3 (50%-75%) and 4 (>75%). The immunoreactivity score (IRS) of each sample was obtained by multiplying staining intensity and staining extent. The expression was classified as high expression if IRS was higher than 6 while IRS of 6 or less indicated low expression.

Supplementary Figure 1

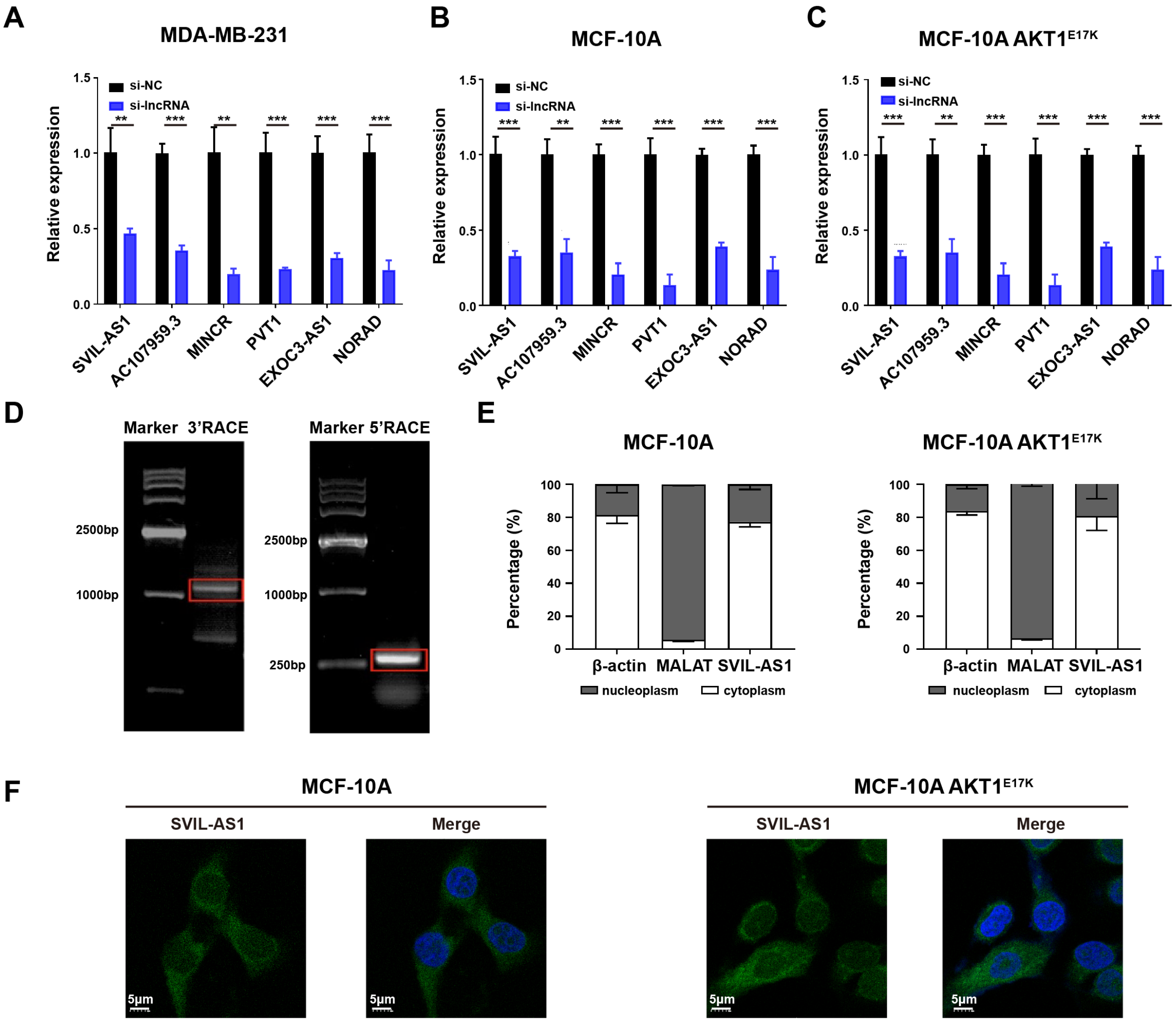

Figure S1. Screening lncRNAs binding to AKT1 E17K mutant protein.

A-C) Knockdown efficiency of lncRNAs in MDA-MB-231, MDA-10A and MDA-10A AKT1^{E17K} cells.

D) 5'- and 3'-RACE of SVIL-AS1 in MCF-10A AKT1^{E17K} cells. Bands in red frames were sent for Sanger sequencing identifications.

E) Subcellular localization of SVIL-AS1 in MCF-10A and MCF-10A AKT1^{E17K} cells, as detected by nuclear and cytoplasmic fraction isolation and RT-qPCR.

F) FISH shows the subcellular localization of SVIL-AS1 in MCF-10A and MCF-10A AKT1^{E17K} cells. Scale bar, 5 μm.

The results are presented as mean ± SD of triplicate experiments. *, $P < 0.05$; **, $P < 0.01$; ***, $P < 0.001$; ****, $P < 0.0001$.

Supplementary Figure 2

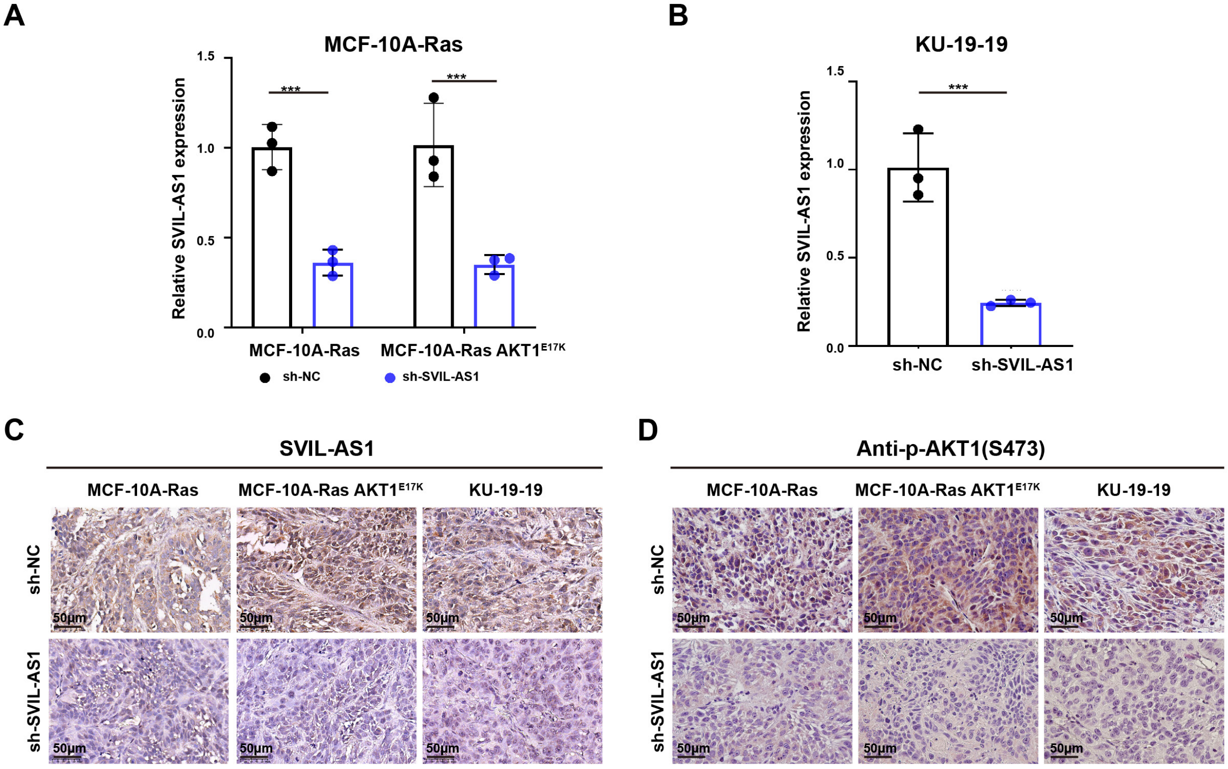

Figure S2. SVIL-AS1 binds to AKT1^{E17K} and enhances AKT1 phosphorylation.

A, **B**) The efficiency of SVIL-AS1 knockdown in xenografts, as confirmed by RT-qPCR.

C) The efficiency of SVIL-AS1 knockdown in xenografts, as confirmed by ISH. Positive ISH signals are detected as brown staining, and nuclei are counterstained with hematoxylin.

D) Representative IHC results of p-AKT1 (S473) in mouse xenografts. Scale bar, 50 μm.

The results are presented as mean ± SD of triplicate experiments. *, $P < 0.05$; **, $P < 0.01$; ***, $P < 0.001$; ****, $P < 0.0001$.

Supplementary Figure 3

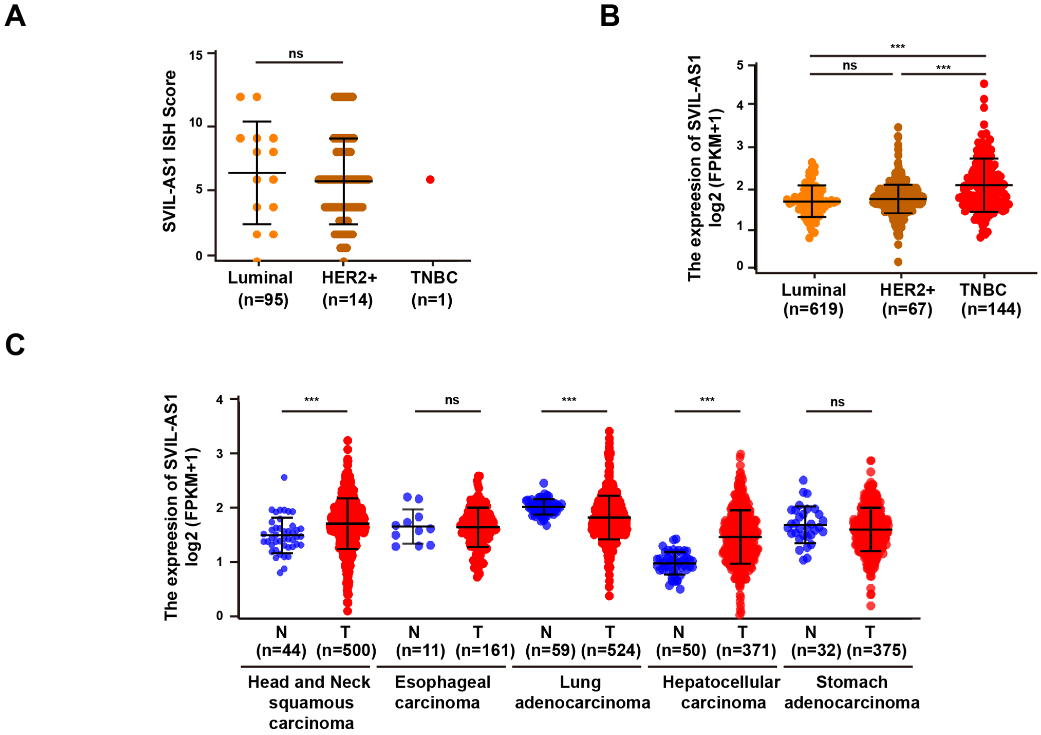

Figure S3. The expression of SVIL-AS1 in tumor tissues.

A) The expression of SVIL-AS1 in breast cancer subtypes in our cohort, as detected by ISH. TNBC, triple negative breast cancer.

B) The expression of SVIL-AS1 in breast cancer subtypes in TCGA dataset. The data show tumor samples with available subtype information.

C) The expression of SVIL-AS1 in normal and tumor tissues in TCGA dataset. N, normal. T, tumor.

The results are presented as mean ± SD. ***, $P < 0.001$; ns, no significant.

Table S1. 5'- and 3'- RACE identify SVIL-AS1 sequence in MCF-10 AKT1^{E17K} cells.

Name	Sequence
**SVIL-AS1**	CTGAATAGCTGAGAGGCGCACCCGGGCGGCCAGCTGGGCTCGGAGTGCA<br>GCGGGGTTAGGATGGACGAGGACGTGTTGCACCCTGAAGAGCCTCATCAT<br>CGCCTGCTCTTGAGGTTCACAGATGATACCTTTGATCCAGAACTTGCAGC<br>AACAATAGATCCATCTTCGGTTGTGAATCCGGCCCGGAATGCAGCCACCC<br>CTTAGAGCAACCCCCCTTGCTGCTCGTGCCCTGCTGTGCTACTGCCTCAGT<br>CACTTCCTGCTTGGTCAAGACCACAGCTGCTGATGTCACAGCTGAGGCAC<br>ATCAGAAAGGCTGCCTGATTCAAAGAAAGCTGCCGGATAAATCATAGAGC<br>ACAGACACTTCTACCTCATGCTCTTATGGGACTTTAGGAACCAAACATAAG<br>CACCTATGGAAAATGCACGGCGGTCTTGAAAGGCTGTGGTCTTTATCAGA<br>AAATATCTGAATGACTGTTTGTTATGTCAGTCCTACTCTGCAGGCTTGCTA<br>AGATGATGATTGAAAGGGCTCTTGGTTTCTGCTTTGGAATCTGGCATTTAT<br>GTTTTGAAGCGTTGTGAGCAAGCGATGTGGCAGATTGCAGGGTGAGGGG<br>GACAAACCTGGGCCAGGAGCCAGGGGTTTCTCTACCCCCTTCCCTGACTT<br>ACAGCTGGAACTGGGCTCAGTGGTTTCAGCCTCATTTGCTAAGAAGGTCT<br>GGGTATGTAATCCTGGCTGCATCTTAAAATCCCTAGGAGCTTTTAAGACAT<br>ATTGACACATAAGCTCTAACTCACATGAAACGAAAACACATCTCTGGGGC<br>TGGGCTGAGCAAATGCATTTTTTAAAAGGCCCCTCAGGTGATTCTGTCTCA<br>GAGATGTTGAAAACCAGTGGGTTAGGAGCTCAGTCATCCACTTTCAGATC<br>TGTGATTATGCCACTATTAAATACAGTTGGCTATCACATAGATTTTATGCTTT<br>AGCTTGTTCTTTTTTCCTTTGCTATACCAAATAAAATCTTCTGAGCTGTAGT<br>TCCAGTCCTTAAAATAATGGTTGTTACCATACTGAGGTTAAACATTAGTGC<br>ATTTGCTTAACAAGGACTTTAAAACTTATCACACTGCCATAATTTTTAATGG<br>TTTCTATAATGATTAATTAATAAGAAAATCAATAATTGGATTTTAAGCAGGA<br>AACTAGGCAGGAAAGTTGAAATTCATAGACCAATTCAATGGAAAACATTA<br>AAATGTATTGGGAGATGCCCAATATTCTATGCCAAAAAAAAAAAAAAAAA<br>AAAAAAAAAAA

Sanger sequencing identified SVIL-AS1 as a 1247 nt transcript which was 4 nt longer in 3' end (labeled in red) than previous reported sequence (Ensemble ID: ENST00000414457).

Table S2. The AKT1-interacting proteins immunoprecipitated with Flag-AKT1 and detected by MS in sh-SVIL-AS1, but not in sh-NC MCF-10A AKT1^{WT} and MCF-10A AKT1^{E17K} cells

MCF-10A AKT1^{WT} cells				MCF-10A AKT1^{E17K} cells			
**Gene Symbol**	**MW [kDa]**	**Coverage [%]**	**Peptides**	**Gene Symbol**	**MW [kDa]**	**Coverage [%]**	**Peptides**
AKT2	81.7	17	10	ATP1A1	112.8	14	13
HIST2H2BE	13.9	12	1	CAMK2A	54.1	3	1
MAP2K1	43.4	3	1	CTNNB1	85.5	12	10
NPM1	32.6	14	2	MARK2	87.9	3	2
PPIA	18	5	1	NSF	82.5	9	6
PPP2R2A	51.7	5	2	PFKP	85.5	2	1
PPP2R5E	54.7	2	1	PPP2R2A	51.7	2	1
				PPP2R5E	54.7	3	1
				RPS6KB2	53.4	2	1

Table S3. The AKT1-interacting proteins immunoprecipitated with Flag-AKT1 and detected by MS in sh-NC, but not in sh-SVIL-AS1 MCF-10A AKT1^{WT} and MCF-10A AKT1^{E17K} cells

MCF-10A AKT1^{WT} cells				MCF-10A AKT1^{E17K} cells			
Gene Symbol	MW [kDa]	Coverage [%]	Peptides	Gene Symbol	MW [kDa]	Coverage [%]	Peptides
BECN1	51.9	2	1	ALDOA	39.4	19	3
CKB	42.6	4	1	BECN1	51.9	2	1
DDX5	69.1	2	1	CKB	42.6	4	1
FLII	144.7	1	2	DDX5	69.1	2	1
MSH2	104.7	4	4	MYH9	226.4	3	4
VIM	53.6	14	7	NPM1	32.6	4	1
VWF	309.1	1	1	PCK2	70.7	2	1
				PPM1A	42.4	3	1
				PPP3CA	58.7	2	1
				TPM4	28.5	3	1

Table S4. The AKT1-interacting proteins detected by MS after RNA pulldown in MCF-10A AKT1^{E17K} cells

Gene Symbol	MW [kDa]	Coverage [%]	Peptides
ASXL1	165.3	1	1
ATP1A1	112.8	14	11
EIF4A1	46.1	12	4
HSPB1	22.8	5	1
LAMA3	190.4	1	2
LAMB3	129.5	7	7
MAPKAP1	59.1	12	6
MARK2	87.9	12	8
NPM1	32.6	3	1
PPP2R1A	65.3	2	1
PPP2R2A	51.7	3	1
PRKAA1	64	2	1
SRPK2	77.5	1	1
VTN	54.3	3	1

Table S5. The siRNA/shRNA sequences of SVIL-AS1

	**Sense（5'-3'）**	**Anti-Sense（5'-3'）**
Sequence 1	GCUACUGCCUCAGUCACUUTT	AAGUGACUGAGGCAGUAGCTT
Sequence 2	GCAAGCGAUGUGGCAGAUUTT	AAUCUGCCACAUCGCUUGCTT

Table S6. Primer sequences for RT-qPCR

Genes	Sense (5'-3')	Antisense (5'-3')
AC016205.1	AGCACCTTTACAGAAGAGGCA	GCCCAGAGGAAAAATAGTGAGC
AC107959.3	GCATTAAATGAGCTCTCAGAGGC	TCATTCTGGGTCACTGCTGG
NORAD	GTGACCACTCTGTCGCCATT	AGAATGAAGACCAACCGCCC
APTR	CGGTGGGTATCAGGGAAGAA	TTCCCGAAATCCAGCTTGCT
EXOC3-AS1	AGATCGAGACCATCCTGGCT	ATCTTGGCTCACTGCAAGCT
FP671120.4	TCACGCTGGTCTCAAACTCC	GAGAAACGTTCACACCGTGC
LINC00839	GACTTTGGAGCGGCCTCATA	GGGTTGCCGTCTTCTCCTTT
MINCR	TTGACAGCTGCAGGTCTTGG	AACCAATGATGTGGGGTGGG
MALAT1	CCTGCAGCTGGTGTTTTGAG	AGTTTTCAGCAGTAGGGCTTCT
PVT1	GACTTCGCAGGTGAGCAGTA	TTGGATAAGGCAGGTGCTGG
SNHG15	TTGAATGCAAGCCTTGGCAC	GGTGCACAGCAAAGCTTCTC
SVIL-AS1	TGAAGAGCCTCATCATCGCC	TTGTTGCTGCAAGTTCTGGA
GAPDH	TCCAAAATCAAGTGGGGCGA	ATGGCATGGACTGTGGTCAT
